# Catalytic core–shell nanoparticles with self-supplied calcium and H_2_O_2_ to enable combinational tumor inhibition

**DOI:** 10.1186/s12951-021-01055-4

**Published:** 2021-10-12

**Authors:** Hanjing Kong, Chao Fang, Qiang Chu, Zefeng Hu, Yike Fu, Gaorong Han, Xiang Li, Yi Zhou

**Affiliations:** 1 State Key Laboratory of Silicon Materials, School of Materials Science and Engineering, Zhejiang University, Hangzhou, Zhejiang 310027 People’s Republic of China; 2 ZJU-Hangzhou Global Scientific and Technological Innovation Center, Zhejiang University, Hangzhou, 311200 People’s Republic of China; 3 Stomatology Hospital, School of Medicine, Zhejiang University, Hangzhou, 310006 People’s Republic of China

**Keywords:** Self-supplied H_2_O_2_, CaO_2_@Co-ferrocene, Calcium overload, ROS generation, Tumor inhibition

## Abstract

Nanoparticles, presenting catalytic activity to induce intracellular oxidative species, have been extensively explored for tumor treatment, but suffer daunting challenges in the limited intracellular H_2_O_2_ and thus suppressed therapeutic efficacy. Here in this study, a type of composite nanoparticles, consisting CaO_2_ core and Co-ferrocene shell, is designed and synthesized for combinational tumor treatment. The findings indicate that CaO_2_ core can be hydrolyzed to produce large amounts of H_2_O_2_ and calcium ions at the acidic tumor sites. Meanwhile, Co-ferrocene shell acts as an excellent Fenton catalyst, inducing considerable ROS generation following its reaction with H_2_O_2_. Excessive cellular oxidative stress triggers agitated calcium accumulation in addition to the calcium ions released from the particles. The combined effect of intracellular ROS and calcium overload causes significant tumor inhibition both in vitro and in vivo.

**Supplementary Information:**

The online version contains supplementary material available at 10.1186/s12951-021-01055-4.

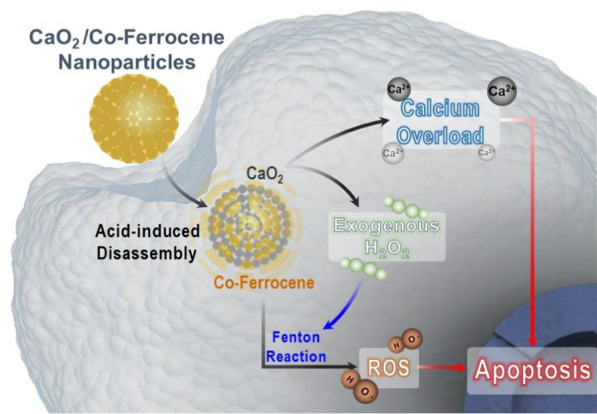

## Introduction

Metal peroxides, including CuO_2_ [1], ZnO_2_ [2], MgO_2_ [3], CaO_2_ [4, 5], and BaO_2_ [6], have been extensively explored for biomedical applications, including tumor therapy. For instance, under an acidic aqueous condition, CaO_2_ can be hydrolyzed to produce Ca^2+^ ions and H_2_O_2_. The excessive content of intracellular Ca^2+^ ions causes cell death via the induction of calcium-overload stress. The recent studies indicate that the uncontrollable accumulation of Ca^2+^ ions in tumor cells may interfere with cell signaling, cause cytotoxicity and trigger apoptosis [7, 8]. In addition, the enrichment of intracellular calcium can also be triggered by the abnormal variation of reactive oxygen species (ROS) [9, 10]. The main mechanism is that ROS regulates the entry and exclusion of calcium ions by affecting proteins of calcium channels or pumps, such as transient receptor potential (TRP) channels [11] and plasma membrane Ca^2+^-ATPase (PMCA) [12]. However, when utilizing CaO_2_ as a potential therapeutic platform for tumor treatment, the strong alkalinity of Ca(OH)_2_, the byproduct of CaO_2_ hydrolyzation, results in severe toxicity to normal cells and tissue. An effective strategy, to lessen its intrinsic toxicity while maintaining its expected antitumoral properties, is therefore highly demanded.

In general, H_2_O_2_ is overexpressed in tumor cells [13], inducing its increased intracellular oxidative stress. Meanwhile, H_2_O_2_ can be converted into highly toxic hydroxyl radicals (·OH) via Fenton reaction triggered by catalytic metal ions, leading to cell apoptosis [14]. This phenomenon, also known as chemodynamic therapy (CDT), has emerged as a highly potential approach for tumor-specific therapy [14, 15]. Even so, the level of intratumoral H_2_O_2_ (~ 50 × 10^–6^ to ~ 100 × 10^–6^ M) remains inadequate to maintain the sustained production of ROS for effective tumor inhibition. A comment strategy is to induce exogenous H_2_O_2_, for example utilizing the glycolysis reactions of glucose oxidase (GOx) [16–18]. As a biocatalyst, GOx can catalyze the oxidation of glucose to supply H_2_O_2_. However, the catalytic reaction is considerably restrained by tumor hypoxia. Thus, CaO_2_ may potentially serve as an alternative H_2_O_2_ sources, due to its strong capability in hydrolysis, to incorporate with CDT agents for promoted tumor inhibition [19].

At present, ferric ion remains as the most comment and efficient catalyst in triggering Fenton reactions, and it is extensively used as an ingredient for the investigation of CDT agents [20]. Ferrocene is an organic transition metallic compound, which is nontoxic under the neutral physiological condition. Centered on Fe (II) with electron donor–acceptor conjugated structure, ferrocene, as a potential heterogeneous Fenton catalyst, represents an excellent redox reversible characteristic [21, 22]. In addition, ferrocene can bridge various metal ions through functional groups modification. It has been recently reported that nanoscale Co-ferrocene MOF possesses enhanced Fenton reactivity, which effectively promote the conversion from H_2_O_2_ to ·OH [23].

**Fig. 1:**
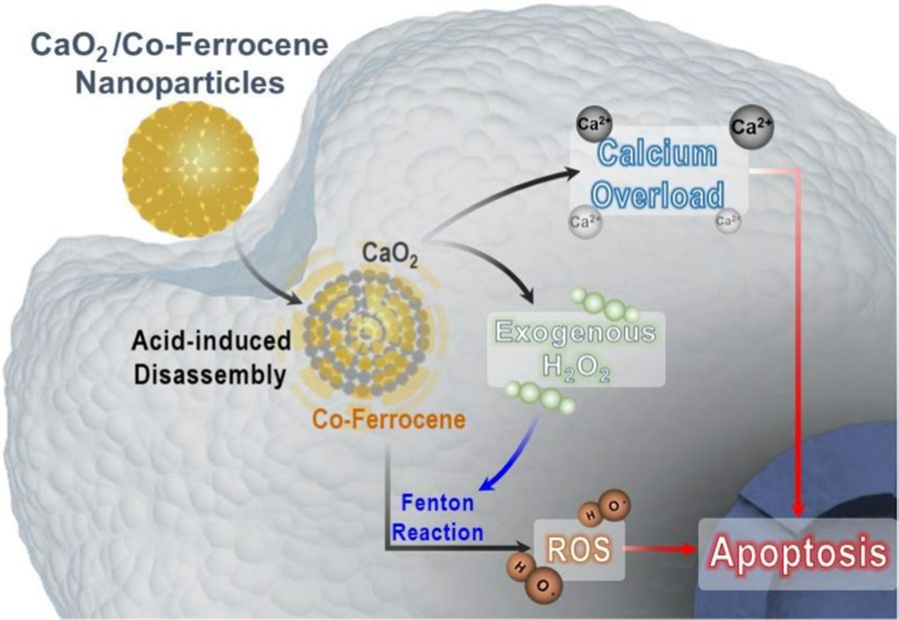
Schematic illustration for the functioning mechanism of CaO_2_@Co-Fc

Herein, a type of composite nanoparticles, presenting unique self-supplied H_2_O_2_, calcium release and Fenton activity, is designed and synthesized for combinational tumor treatment with both tumor specificity and therapeutic efficacy (Fig. 1). In this system, CaO_2_ nanoparticles are covered and protected with a Co-ferrocene shell (denoted as CaO_2_@Co-Fc). The findings indicate that CaO_2_@Co-Fc may remain stable in the neutral aqueous condition, and hydrolyze to produce H_2_O_2_ and release Ca ions due to the degradation of Co-Fc shell in the acidic TME. The H_2_O_2_ reacts with Co-Fc molecules to generate considerable content of cytotoxic ·OH. More interestingly, the induction of intracellular ROS further agitates the intracellular aggregation of Ca^2+^ ions, leading to calcium overload. The combined effects of strong CDT phenomenon mediated by self-supplied H_2_O_2_ and calcium overload in cancer cells enable significant anticancer properties both in vitro and in vivo.

## Materials and methods

### Chemicals and agents

Calcium chloride anhydrous (CaCl_2_, AR), hydrogen peroxide (H_2_O_2_, 30 wt%), ammonia solution (NH_3_·H_2_O, 25 wt%), N, N-dimethylformamide (DMF, AR), and ethanol anhydrous were purchased from Sinopharm Chemical Reagent Co. Ltd. Cobalt (II) acetate tetrahydrate ((CH_3_COO)_2_Co·4H_2_O, 99.9%) and 1,1’-Ferrocenedicarboxylic acid (Fc(COOH)_2_, > 98%) were purchased from Aladdin-Reagent Co. Ltd. Polyvinyl pyrrolidone (PVP, K30), Fluo-4 AM and Cell Count Kit-8 (CCK-8) were purchased from Meilunbio Co. Ltd. 3, 3’, 5, 5’-Tetramethylbenzidine (TMB), 5, 5-Dimethyl-1-pyrroline N-oxide (DMPO), 2’,7’-Dichlorofluorescin diacetate (DCFH-DA, ≥ 97%) were purchased from Sigma-Aldrich. Horseradish peroxidase (HRP) was purchased from Hefei Bomei Biotechnology. The ECL western blotting system was purchased from Beyotime Biotechnology. Primary and secondary antibodies were obtained from Abcam. Calcein AM and propidium iodide (PI) probes were obtained from Dojindo.

### Characterization

Scanning electron microscopy (SEM) images was obtained by field-emission scanning electron microscopy (FESEM, Phenom Pharos). Transmission electron microscopy (TEM) images was obtained by transmission electron microscopy (TEM, HITACHI HT-7700; X-MAX^n^65 T). The zeta potential and dynamic light scattering were measured by zetasizer (0.3–10,000 nm, Zetasizer Nano-ZS Malvern). The X-ray diffraction pattern was obtained by X-ray diffractometer with Cu Kα radiation (XRD, X’pert PRO MPD). The chemical composition and valence states of different elements were measured by X-ray photoelectron spectroscopy (XPS, AXIS Supra). The UV–visible absorbance was measured by UV–vis spectrophotometer (Shimadzu, UV-2600). Hydroxyl radical signal was monitored by electron spin resonance spectroscopy (EPR, Bruker A300). CCK-8 and intracellular H_2_O_2_ content were recorded using a microplate reader. The fluorescence images of Live and Dead, intracellular reactive oxygen species (ROS) and Ca^2+^ ions were obtained by an inverted fluorescent microscope (Nexcope, The USA).

### Synthesis of CaO_2_@Co-ferrocene

CaO_2_ nanoparticles were prepared according to previous procedures with the following protocol: CaCl_2_ (0.1 g) and PVP (0.35 g) were dissolved in 15 mL ethanol. Subsequently, NH_3_·H_2_O (0.8 M, 1 mL) was added under continuously stirring. Then, H_2_O_2_ (1 M, 0.2 mL) was added dropwise to the mixture using a syringe pump. Finally, CaO_2_ nanoparticles were collected by centrifugation at 12,000 rpm for 10 min and washed three times with ethanol. The product was stored in 5 mL ethanol for further use. CaO_2_@Co-ferrocene was prepared via in situ assembly. Typically, CaO_2_ (5.3 mg) and PVP (160 mg) were dissolved into 18 mL DMF under ultrasound treatment. Afterwards, (CH_3_COO)_2_Co·4H_2_O (0.1 M, 0.2 mL) and Fc(COOH)_2_ (0.1 M, 0.2 mL) were added. After stirring at 80 °C for 4 h, the product was obtained by centrifugation and washed several times with DMF and ethanol, respectively.

### H_2_O_2_ generation

In brief, CaO_2_@Co-ferrocene (0.5 mg, or CaO_2_ containing the same amount of Ca^2+^) was dissolved in 0.5 mL acetate buffer solution (ABS) at pH 5 or 7. Then, 20 μL supernatant was collected at the different time interval. HRP (1 U/mL, 300 μL) and phosphate buffer solution (pH = 5.8, 1.68 mL) were added. After 10 min’ reaction, TMB (2 mM, 300 μL) was added and the characteristic absorbance of ox-TMB at 371 nm was recorded to quantify the H_2_O_2_ concentration according to the standard curve.

### Chemodynamic activity

3, 3’, 5, 5’-Tetramethylbenzidine (TMB) can be oxidized to the oxidation state of TMB by ·OH produced by CaO_2_@Co-ferrocene under acidic condition, which shows a characteristic UV–vis absorbance at 655 nm. In brief, different concentrations of CaO_2_@Co-ferrocene and TMB (8 mM, 300 μL) were added in ABS at different pH (5 and 7). The UV–vis absorption of various samples was recorded by UV–vis spectrophotometer. Subsequently, the reaction kinetic curve was monitored under a UV–vis spectrophotometer. The generation of ·OH was further identified by electron spin resonance (ESR) spectroscopy with 5, 5-Dimethyl-1-pyrroline N-oxide (DMPO) as a spin trap. In addition, CaO_2_@Co-ferrocene (1 mg, or CaO_2_ with the same amount of Ca^2+^) was dissolved in ABS (initial pH = 5, 2 mL). pH value was recorded with a real-time pH meter.

### In vitro study

Human liver cell (7702), human umbilical vein endothelial cell (HUVEC) and murine breast cancer (4T1) cells were used for the in vitro study. The culture medium RPMI 1640 was supplemented with 10% fetal bovine serum (FBS) and the cells were cultured in an incubator with 5% CO_2_ at 37 °C.

#### Cell viability assay

7702 and HUVEC cells were seeded in 96-well plates (10^4^ cells per well) and incubated for 12 h. Subsequently, CaO_2_@Co-ferrocene at desired concentrations were added and incubated for another 24 h. Finally, culture medium containing 10% CCK-8 was added to each well and the absorbance was detected by a microplate reader at 450 nm after incubation at 37 °C for another 1 h.

#### Intracellular antitumor performance

4T1 cells were seeded in 96-well plates (10^4^ cells per well). After incubation for 12 h, the culture medium was replaced with fresh medium at different pH values. To simulate acidic tumor microenviroment, the pH of cell culture was regulated to 6.5. HCl solution (1 M, 15 μL) was added into the medium (980 μL). The acidic medium was added along with CaO_2_@Co-ferrocene. Then, CaO_2_@Co-ferrocene at desired concentrations were added and incubated for another 24 h. Finally, culture medium containing 10% CCK-8 was added to each well and the absorbance was detected by a microplate reader at 450 nm after incubation at 37 °C for another 1 h.

In addition, the live & dead cells staining was carried out using calcein AM/PI staining. After seeded in 6-well plate and cultured for 12 h, 4T1 cells were cultured with CaO_2_@Co-ferrocene at desired concentrations for 10 h. After treatments, cells were cultured with calcein AM and PI for 30 min. After staining, cells were washed with PBS for three times and further observed at 480 nm and 525 nm, respectively.

#### Colony efficiency assay

4T1 cells were seeded in 6-well plates (200 cells per well). After incubation for 12 h, the cells were cultured in fresh medium containing different concentrations of CaO_2_@Co-ferrocene for 10 days. Then, 4T1 cells were fixed in 10% formaldehyde after washed with PBS. Finally, 4T1 cells were stained by crystal violet for 20 min.

#### Examination of intracellular calcium, ROS and H_*2*_*O*_*2*_

ROS was detected by a fluorescent probe, 2, 7- dichlorofluorescein diacetate (DCFH-DA). 4T1 cells were seeded in 6-well plates. After incubation for 12 h, the cells were cultured in fresh medium containing different concentrations of CaO_2_@Co-ferrocene for another 24 h. Then, the medium was discarded, and the cells were washed three times with PBS before the addition of DCFH-DA (2 mg/mL, 10 μL). After staining, cells were washed with PBS for three times and DCF fluorescence images were observed by an inverted fluorescent microscope at an excitation wavelength of 480 nm. To detect intracellular Ca^2+^ ions, Fluo-4 AM, was applied as a green fluorescent probe according to the above steps. After treatments with CaO_2_@Co-ferrocene (60 μg/mL), 4T1 cells were lysed by RIPA lysis buffer with 1% PMSF. Supernatant containing H_2_O_2_ was collected by centrifugation at 12,000 rpm. Then, hydrogen peroxide assay kit was added and the absorbance at 560 nm was recorded with microplate reader.

#### Western blot assay

After treatments with CaO_2_@Co-ferrocene, 4T1 cells were lysed by RIPA lysis buffer with 1% PMSF. The collected cells were further crushed with an ultrasonic probe. Supernatant containing proteins was collected by centrifugation at 12,000 rpm. After quantification, protein (50 μg) was loaded onto SDS-PAGE, further transferred to the PVDF membrane and blocked by 5% skim milk for 2 h. Then, the PVDF membrane was incubated with primary antibodies and secondary antibodies successively. After washing three times in Tris-buffered saline Tween buffer (TBST), the band intensity was measured.

### In vivo study

Four-week-old female Balb/c mice were purchased from Shanghai Slac Laboratory Animal Co. Ltd. and used in compliance with guidelines of the Biological Resource Centre of the Agency for Science, Technology and Research, Zhejiang University. The tumor-bearing mouse model was built via injecting 50 μL PBS with 4T1 cells (1 × 10^6^) into the right side back of each mouse. The mice were randomly distributed into four groups for in vivo experiments (5 mice per group) when the tumor volumes reached about 80 mm^3^ and intratumorally injected with different formulations: Group 1: PBS, Group 2: Co-Fc MOF, Group 3: 5 mg/kg CaO_2_@Co-Fc, Group 4: 15 mg/kg CaO_2_@Co-Fc. The tumor size (V) was calculated as follows: V = width^2^ × length/2. Body weight, tumor size and images were recorded every 2 days. After 2 weeks, tumors were collected for immunohistochemistry analysis after sacrifice.

The paraffin-embedded slices were deparaffinized in xylene and subsequently hydrated in serially diluted grades of ethanol. Endogenous peroxidase was blocked with 3% hydrogen peroxide, followed by antigen retrieval using a microwave oven under at pH = 6.0 (citrate buffer). Slices were incubated with ki67 overnight at 4 °C and then overlaid with secondary antibody for 20 min at room temperature. Finally, a diaminobenzidine tetrahydrochloride (DAB) working solution was applied and the slices were counterstained with hematoxylin.

### Statistical analysis

Data were expressed as mean ± SD. Comparison analysis between groups was conducted by student’s test.

## Results and discussion

### Synthesis of CaO_2_@Co-Fc

**Fig. 2:**
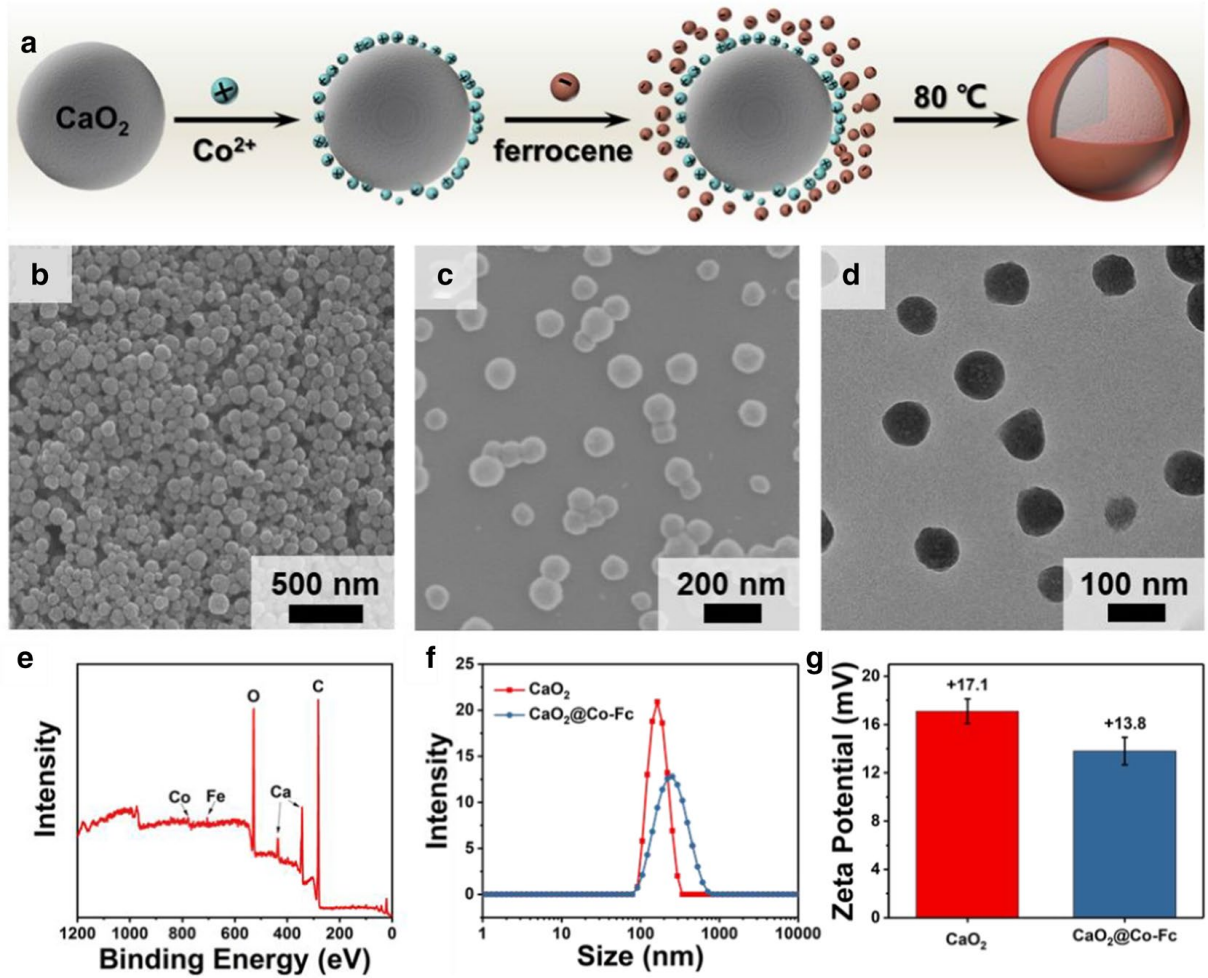
**a** Schematic illustration of the synthesis procedure of CaO_2_@Co-Fc. **b** SEM image of CaO_2_. **c** SEM image of CaO_2_@Co-Fc. **d** TEM image of CaO_2_@Co-Fc. **e** XPS survey spectra of CaO_2_@Co-Fc. **f** Size distribution of CaO_2_and CaO_2_@Co-Fc. **g** Zeta potential of CaO_2_ and CaO_2_@Co-Fc

As illustrated in Fig. 2a, CaO_2_ was firstly obtained through a typical wet-chemistry approach following the approach reported previously [24]. Subsequently, Co^2+^ ions and ferrocene molecules were absorbed at the surface of CaO_2_ nanoparticles. Under the heating at 80 ℃, the Co-Fc coating was formed. As shown in the scanning electron microscopy (SEM, Fig. 2b) and transmission electron microscopy (TEM, Additional file 1: Fig. S1) images, CaO_2_ particles formed are of spherical morphology with rough surface and uniform dimension of ~ 80 nm. After the formation with Co-Fc shell, the composite nanoparticles possess a gently increased size, and the surface becomes smoother (Fig. 2c, d). the analysis using X-ray photoelectron spectroscopy (XPS) reveals that the composite particles present the peaks of Co and Fe elements, confirming the existence of Co-Fc coordination compound, and the particle solution turns from white into yellow colour (Fig. 2e, Additional file 1: Fig. S2). The high resolution XPS spectra verify that the main valence state of iron element is divalent, and partial oxygen exists as peroxo groups (Additional file 1: Fig. S3). As shown in Fig. 2f, hydrodynamic particle size increases after being coated by Co-Fc, which is consistence with the findings from SEM and TEM examinations. Due to the negatively charged Co-Fc compound (Co-Fc MOF), the surface potential decreases from + 17.1 mV to + 13.8 mV (Fig. 2g). The coating of Co-Fc compound on CaO_2_ nanoparticles is also verified by the characterization using energy dispersive spectrometer (EDS, Additional file 1: Fig. S4). Co and Fe elements are evenly distributed at the surface of CaO_2_ nanoparticles. The X-ray diffraction (XRD) pattern of CaO_2_@Co-Fc corresponds to the characteristic peaks belonged to CaO_2_ (PDF#03-0865, Additional file 1: Fig. S5). There are no characteristic peaks of Co-Fc MOF observed, indicating that the Co-Fc shell is in an amorphous nature.

### Functional characteristics of CaO_2_@Co-Fc

**Fig. 3:**
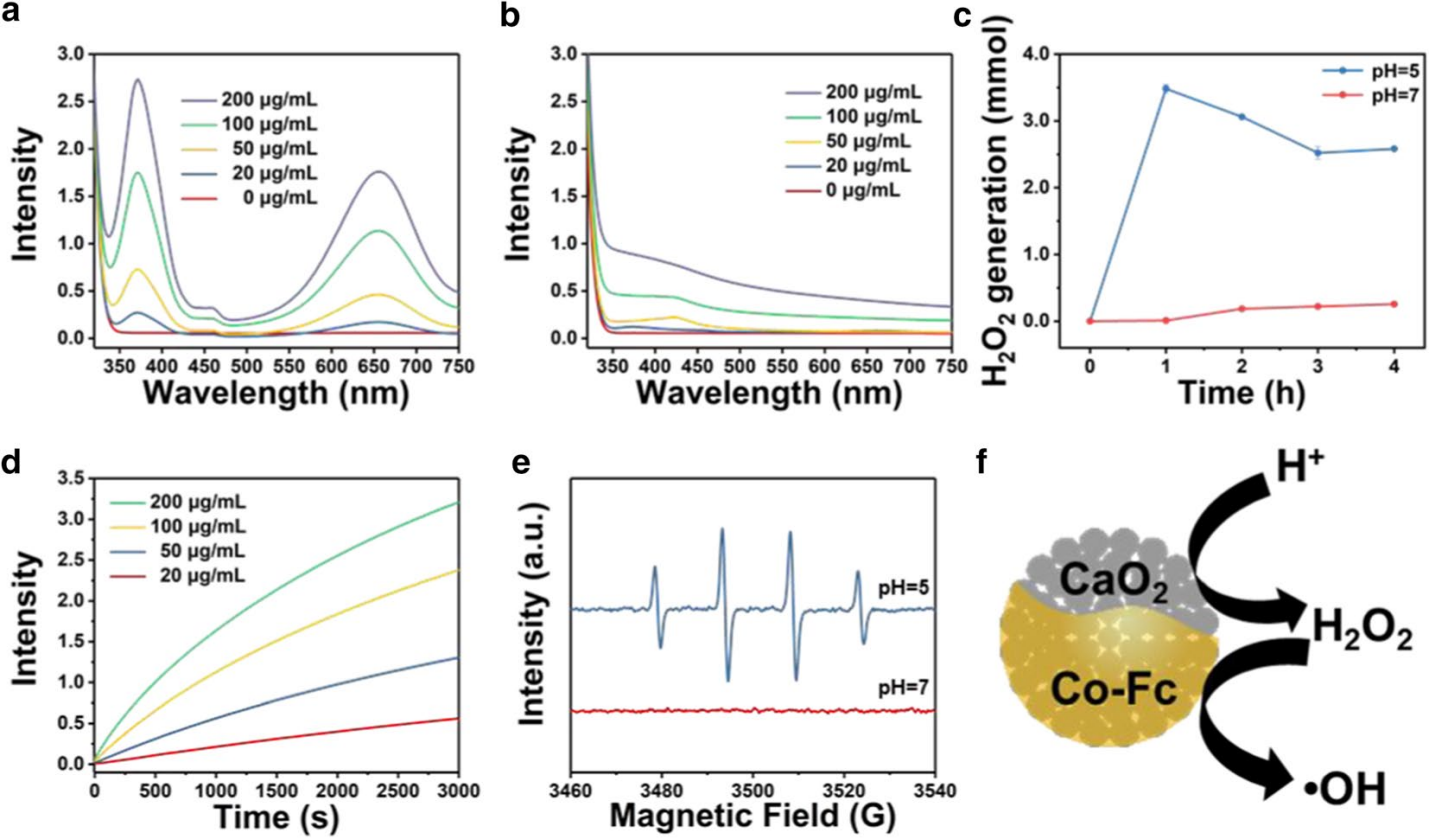
UV–vis spectra of CaO_2_@Co-Fc with varied concentrations in TMB solutions of (**a**) pH 5 and (**b**) pH 7. **c** H_2_O_2_ release profile of CaO_2_@Co-Fc at different pH values. **d** Time-course absorbance at 655 nm with different concentrations of CaO_2_@Co-Fc in TMB solution (**e**) EPR analysis of ·OH production of CaO_2_@Co-Fc at different pH values. **f** Schematic illustration of the in vitro performance of CaO_2_@Co-Fc

During the characterization, 3, 3’, 5, 5’-Tetramethylbenzidine (TMB) chromogenic method was used to evaluate the Fenton activity of as-prepared CaO_2_@Co-Fc, due to the fact that TMB can be oxidized by ·OH to give an aquamarine blue color with characteristic absorbance at about 650 nm [18, 25]. As shown in Fig. 3a, the characteristic absorption peaks of ox-TMB are observed, accompanying with the distinct color change under an acidic condition. In addition, the rate of the chromogenic reaction accelerates with the increased concentration of CaO_2_@Co-Fc, showing a typical concentration-dependent manner. In contrast, under neutral condition, no clear absorption can be detected at the same peak position (Fig. 3b), indicating that the Fenton reaction of CaO_2_@Co-Fc occurs in a selective manner corresponding to the acidic condition. The generation of H_2_O_2_ from the particles was examined at different pH values, as shown in Fig. 3c and Additional file 1: Fig. S6_._ No clear sign regarding the H_2_O_2_ generation from CaO_2_@Co-Fc is observed under the neutral condition, and in contrast H_2_O_2_ can be generated rapidly when the pH of solution is varied to 5. It is noteworthy that the content of H_2_O_2_ generated increases at the early stage and decreases after reaching the peak magnitude. The potential reason can be that the dissociation of CaO_2_@Co-Fc under the acidic condition induces agitated production of H_2_O_2_ initially, and that is consumed by Co-Fc molecules, leading to a drop in the H_2_O_2_ content in the solution. During the consumption of H_2_O_2_, the absorbance of TMB solution at ~ 655 nm is enhanced over time, indicating that the ROS could be produced sustainably (Fig. 3d). It is clear that the ROS production is significantly promoted with the increased concentration of CaO_2_@Co-Fc. As indicated by the analysis using electron paramagnetic resonance (EPR), when CaO_2_@Co-Fc is immersed in an acidic solution for a certain period, a typical four-fold peak is observed, confirming the ROS generated belongs to hydroxyl radicals (·OH), and this is attributed to the Fenton catalytic reaction by the H_2_O_2_ generated and Co-Fc molecules (Fig. 3e). Overall, the as-prepared CaO_2_@Co-Fc can effectively trigger the cascaded hydrolysis and catalytic reactions. As depicted in Fig. 3f, once CaO_2_@Co-Fc is exposed to the acidic condition, the Co-Fc shell is disintegrated, and the CaO_2_ core is hydrolyzed to produce a considerable amount of H_2_O_2_. The generated H_2_O_2_ serves as the substrate for the following Fenton reaction triggered by Co-Fc molecules, inducing the highly active hydroxyl radicals.

### In vitro study

**Fig. 4:**
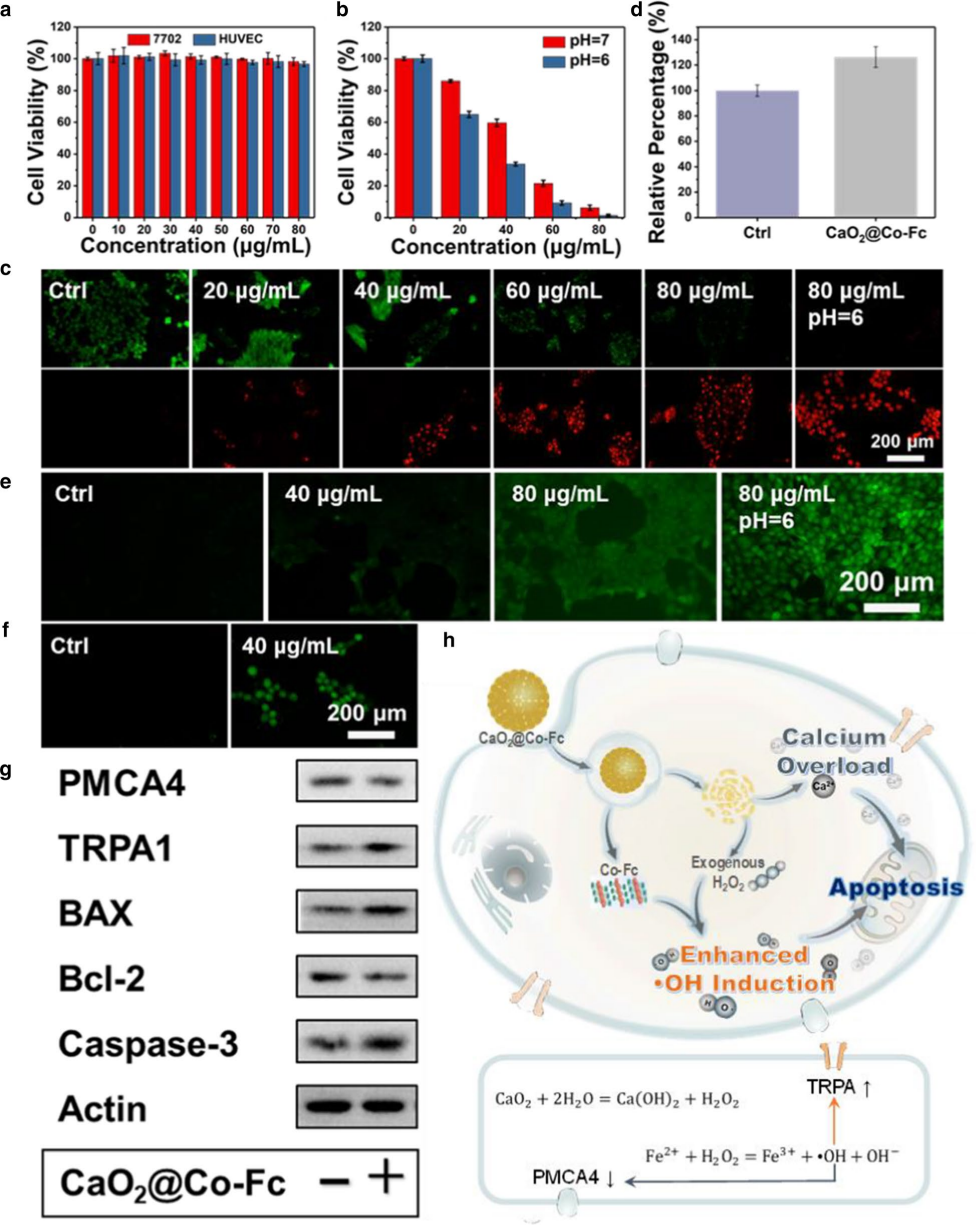
**a** The viability of 7702 and HUVEC cells incubated with CaO_2_@Co-Fc with varied concentrations. **b** Viability of 4T1 tumor cells treated with different concentrations of CaO_2_@Co-Fc. **c** Fluorescence images of calcein-AM and propidium iodide (PI) stained 4T1 cells treated with different concentrations of CaO_2_@Co-Fc. **d** Intracellular H_2_O_2_ content in 4T1 cells after being treated with CaO_2_@Co-Fc. Fluorescence images of (**e**) ROS level and (**f**) Ca^2+^ ions in 4T1 cells with different treatments. **g** The expression of PMCA4, TRPA1, BAX, BCl-2 and caspace-3 in 4T1 cells after treated with CaO_2_@Co-Fc. **h** Schematic illustration for the intracellular functioning mechanism of CaO_2_@Co-Fc

Before evaluating its anti-tumor effect, its cytocompatibility of CaO_2_@Co-Fc was investigated using two types of normal cell lines. As shown in Fig. 4a, CaO_2_@Co-Fc shows negligible cytotoxicity toward human liver cells (7702) and human umbilical vein endothelial cells (HUVEC) at the concentration range of 0–80 μg/mL. In contrast, pure CaO_2_ nanoparticles cause clear negative effect to the viability of 7702 normal cells (Additional file 1: Fig. S7). After being incubated with breast cancer cells (4T1) for 24 h, 20 μg/mL CaO_2_@Co-Fc could cause a certain magnitude of inhibition effect, and this is enhanced with the increase of particle concentration (Fig. 4b). It is noteworthy that the killing effect of CaO_2_@Co-Fc is significantly enhanced under the acidic condition. This is mainly attributed to that acidic condition does not only promote the degradation of nanoparticles and the H_2_O_2_ production [26], but also provides a favoring condition for the ·OH induction. The live & dead assay verifies that CaO_2_@Co-Fc exhibits clear killing effect to tumor cells in both concentration- and pH-dependent manner (Fig. 4c and Additional file 1: Fig. S8). In addition, the inhibitory effect of CaO_2_@Co-Fc on the proliferation of tumor cells was confirmed by colony formation assay (Additional file 1: Fig. S9).

To explore the inhibition mechanism of CaO_2_@Co-Fc to tumor cells, a variety of intracellular indices were characterized. As shown in Fig. 4d, the H_2_O_2_ content presents higher magnitude in the tumor cells incubated with CaO_2_@Co-Fc, indicating the effective H_2_O_2_-supply by the particles. Compared with the control group (PBS), H_2_O_2_ content is ~ 26% higher in cells treated with CaO_2_@Co-Fc. In addition, the intracellular ROS was also be detected using 2,7-Dichlorodi-hydrofluorescein diacetate (DCFH-DA, an ROS probe). The intracellular ROS fluorescence images show that the CaO_2_@Co-Fc-treated cells present bright fluorescence (Fig. 4e and Additional file 1: Fig. S10), indicating that the nanoparticles can be effectively uptaken and produce ·OH in the tumor cells. The fluorescent intensity increases with the increase of the CaO_2_@Co-Fc concentration. Notably, the acidic condition can significantly promote the intracellular ROS fluorescence, suggesting tremendous ROS induction at a lower pH value. The content of intracellular Ca^2+^ ions was examined by calcium fluorescent probe (Fluo-4 AM). As shown in Fig. 4f and Additional file 1: Fig. S11, strong green fluorescence could be observed in CaO_2_@Co-Fc-treated cells compared with the control group, indicating that CaO_2_@Co-Fc could be effectively disintegrated in 4T1 cells and generate significant Ca^2+^ aggregation. Western blot assay was used to investigate the underlying interaction behind these phenomena. In general, the surges of ROS and Ca^2+^ ions are intrinsically avoided by tumor cells to prevent apoptosis [27]. Redox regulation of calcium homeostasis can occur via oxidation of Ca^2+^ channels or its regulators. For example, high levels of oxidative stress can inhibit cytoplasmic Ca^2+^ extrusion through the downregulation of plasma membrane Ca^2+^ ATPase [28] (PMCA, a plasma membrane Ca^2+^efflux pumps). Meanwhile, transient receptor potential (TRP) channels can enhance Ca^2+^ entry [11]. As shown in Fig. 4g, the expression of PMCA4 is reduced whereas that of TRPA1 is strengthened after being incubated with CaO_2_@Co-Fc, indicating that nanoparticles induce considerable influence on the activity of Ca^2+^ channel-related protein by inducing oxidative stress. Mitochondria and endoplasmic reticulum play a crucial role in shaping the Ca^2+^ signal. When intracellular calcium surges, as the outcomes of calcium overload, the ER stress and mitochondrial dysfunction arise (Additional file 1: Fig. S12), which could further activate apoptosis-associated pathway, as confirmed by the alterations in the expression of BAX, Bcl-2 and Caspase-3.

Therefore, the intracellular phenomena induced by CaO_2_@Co-Fc is clear, As demonstrated in Fig. 4h, after being exposed to acidic tumor environment, the nanoparticles disintegrate, and the internal CaO_2_ is hydrolyzed to produce a large amount of H_2_O_2_. In the presence of Co-Fc, H_2_O_2_, both endogenous and exogenous, reacts with Fe ions of Co-Fc molecules to induce considerable production of toxic ·OH, intensifying the cellular oxidative stress. The Co-Fc shell does not only act as Fenton catalyst, but also served as the protection to the CaO_2_ core. In addition, high level of oxidative stress induces intracellular calcium accumulation by regulating calcium channels in the cell membrane, especially TRPA and PMCA4. The excessive ROS and calcium overload occurred within cells eventually activate the apoptosis pathway and lead to cell death [29].

### In vivo anti-tumor properties

**Fig. 5:**
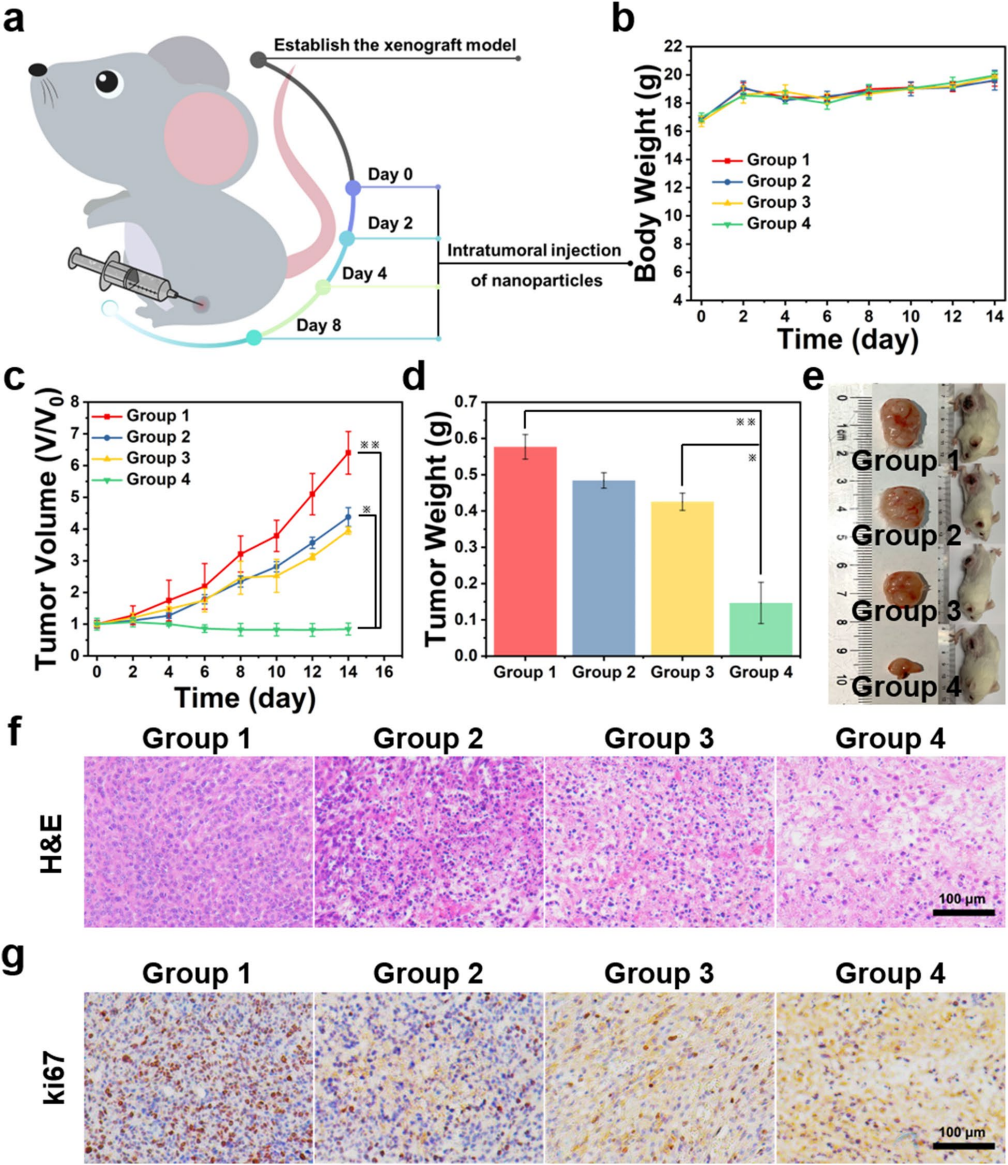
**a** Experimental procedures of injection treatment. **b** Body weight and **c** relative cancer volume variations of 4T1 tumor-bearing mice after different treatments within 14 days. **d** Weight and **e** representative photos of the tumors collected from different groups at 14th day. **f** H&E and **g** ki67 stained tumor slices collected from different groups at 14th day. (Group 1: PBS, Group 2: Co-Fc MOF, Group 3: 5 mg/kg CaO_2_@Co-Fc, Group 4: 15 mg/kg CaO_2_@Co-Fc.)

The anti-tumor efficacy of CaO_2_@Co-Fc was examined using 4T1 tumor-bearing Balb/c mice. The mice were randomly divided into four groups (n = 5) with different treatments (Group 1: PBS; Group 2: Co-Fc MOF; Group 3: 5 mg/kg CaO_2_@Co-Fc; Group 4: 15 mg/kg CaO_2_@Co-Fc), and treated via intratumoral injection (Fig. 5a). There is no significant variation in body weight in the following 14 days, suggesting the negligible side effects of the treatments to mice (Fig. 5b). According to the variation tendency of tumor volume between different groups of mice, CaO_2_@Co-Fc nanoparticles has induced the most intense tumor inhibition (Fig. 5c, Additional file 1: Fig. S13). In contrast, Co-Fc MOF manifests the limited inhibitory effect on tumor growth, which is potentially attributed to its sole CDT effect. Compared with Co-Fc MOF, the integration of CaO_2_ dramatically improves its tumor inhibitory effect, as expected. With the increase of injection dose, the antitumor effect of the CaO_2_@Co-Fc nanoparticles is enhanced, suggesting a dose-dependent tumor inhibition manner. The tumor weight (Fig. 4d) and tumor photograph (Fig. 5e) on day-14 also verifies the tumor regression effect of CaO_2_@Co-Fc nanoparticles. The corresponding H&E staining images of tumor slices visually show the tumor damage between different groups. As shown in Fig. 5f, the tumor tissues treated with CaO_2_@Co-Fc nanoparticles possess typical apoptotic characteristics such as vacuolation, nuclear shrinkage and cell membrane rupture. Meanwhile, Ki67 stained images present similar results with the decreasing positive cells, confirming the proliferation inhibition of CaO_2_@Co-Fc nanoparticles (Fig. 5g). The findings indicate that CaO_2_@Co-Fc nanoparticles present cascaded Fenton activity and enable the excellent in vivo anti-tumor efficacy.

## Conclusions

In this study, a type of nanoparticles with core–shell microstructure, presenting unique self-supplied H_2_O_2_, calcium release and Fenton activity, is designed and synthesized for combinational tumor treatment with both therapeutic specificity and efficacy. In this system, fine CaO_2_ nanoparticles are covered and protected with a Co-ferrocene shell. The findings indicate that CaO_2_@Co-Fc remains stable in the neutral aqueous condition, and hydrolyzes to produce H_2_O_2_ and release Ca ions due to the degradation of Co-Fc shell in the acidic TME. The H_2_O_2_ reacts with Co-Fc molecules to generate considerable content of cytotoxic ·OH. More interestingly, despite the calcium ions released from the particles intracellularly, the induction of intracellular ROS further agitates the intracellular aggregation of Ca^2+^ ions, leading to considerable cellular calcium overload. The combined effects of strong ·OH induction mediated by self-supplied H_2_O_2_ and calcium overload in cells enable significant tumor inhibition both in vitro and in vivo. Overall, this study appears to offer a therapeutic platform with alternative concept, featuring self-supplied H_2_O_2_ and calcium overload, for effective combinational tumor treatment.

## Supplementary Information

**Additional file 1: Fig. S1.** TEM image of CaO_2_. **Fig. S2**. Optical images of solutions containing (a) CaO_2_ and (b) CaO_2_@Co-Fc. **Fig. S3.** High-resolution XPS spectra of (a) Co 2p, (b) Fe 2p and (c) O 1 s. **Fig. S4**. Elemental mapping of CaO_2_@Co-Fc. **Fig. S5**. XRD pattern of CaO_2_@Co-Fc. **Fig. S6**. Standard curves of H_2_O_2_ at the peak of 372 nm by TMB method: (a) UV–vis absorbance spectra and (b) plotting curve of TMB solution with the addition of different concentrations of H_2_O_2_. **Fig. S7**. The viability of 7702 cells cultured with CaO_2_ with varied concentrations. **Fig. S8**. Bright field images of 4T1 cells incubated with different concentrations of CaO_2_@Co-Fc after calcein-AM and PI staining for live & dead. **Fig. S9.** Colony formation of 4T1 cells incubated with different concentrations of CaO_2_@Co-Fc. **Fig. S10**. Bright field images of 4T1 cells incubated with different treatments after DCFH-DA staining for intercellular ROS. **Fig. S11.** Bright field images of 4T1 cells incubated with or without CaO_2_@Co-Fc after Fluo-4 AM staining for intercellular Ca^2+^ accumulation.

## Data Availability

The datasets used and/or analysed during the current study are available from the corresponding author on reasonable request.
