# Supplementary material for: Catalytic core–shell nanoparticles with self-supplied calcium and H2O2 to enable combinational tumor inhibition
Source: J Nanobiotechnology. 2021 Oct 12;19:313. doi: 10.1186/s12951-021-01055-4 (PMC8507391; doi:10.1186/s12951-021-01055-4)
Supplement: Supplementary file 1 — Additional file 1: Fig. S1. TEM image of CaO2. Fig. S2. Optical images of solutions containing (a) CaO2 and (b) CaO2@Co-Fc. Fig. S3. High-resolution XPS spectra of (a) Co 2p, (b) Fe 2p and (c) O 1 s. Fig. S4. Elemental mapping of CaO2@Co-Fc. Fig. S5. XRD pattern of CaO2@Co-Fc. Fig. S6. Standard curves of H2O2 at the peak of 372 nm by TMB method: (a) UV–vis absorbance spectra and (b) plotting curve of TMB solution with the addition of different concentrations of H2O2. Fig. S7. The viability of 7702 cells cultured with CaO2 with varied concentrations. Fig. S8. Bright field images of 4T1 cells incubated with different concentrations of CaO2@Co-Fc after calcein-AM and PI staining for live & dead. Fig. S9. Colony formation of 4T1 cells incubated with different concentrations of CaO2@Co-Fc. Fig. S10. Bright field images of 4T1 cells incubated with different treatments after DCFH-DA staining for intercellular ROS. Fig. S11. Bright field images of 4T1 cells incubated with or without CaO2@Co-Fc after Fluo-4 AM staining for intercellular Ca2+ accumulation. [file 12951_2021_1055_MOESM1_ESM.docx]

**Additional file 1**

**Catalytic Core-shell Nanoparticles with Self-supplied Calcium and H_2_O_2_ to enable Combinational Tumor Inhibition**

*Hanjing Kong,*^1, #^, *Chao Fang,*^1, #^ *Qiang Chu,*^1, 2^ *Zefeng Hu,*^1^ *Yike Fu,^2^ Gaorong Han,*^1^ *Xiang Li*^1, 2^ * *and Yi Zhou,^3,^**

^1^ State Key Laboratory of Silicon Materials, School of Materials Science and Engineering, Zhejiang University, Hangzhou 310027, P.R. China

^2^ ZJU-Hangzhou Global Scientific and Technological Innovation Center, Zhejiang University, Hangzhou, 311200, P.R. China.

^3^ Stomatology Hospital, School of Medicine, Zhejiang University, 310006, P.R. China

**^#^** Authors with equal contribution

* Corresponding author: xiang.li@zju.edu.cn (XL); syuthscsa@zju.edu.cn (YZ)


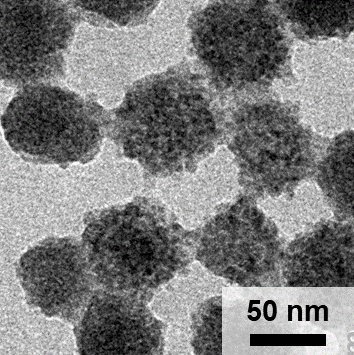


**Figure S1** TEM image of CaO_2_.


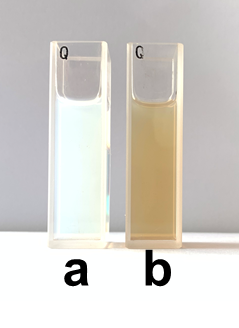


**Figure S2** Optical images of solutions containing (a) CaO_2_ and (b) CaO_2_@Co-Fc.


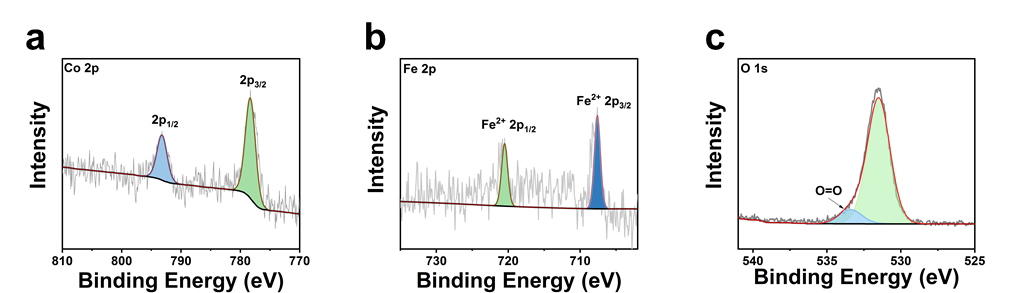


**Figure S3** High-resolution XPS spectra of (a) Co 2p, (b) Fe 2p and (c) O 1s.


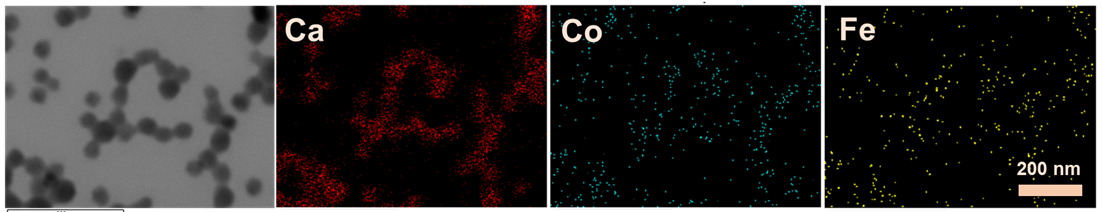


**Figure S4** Elemental mapping of CaO_2_@Co-Fc.


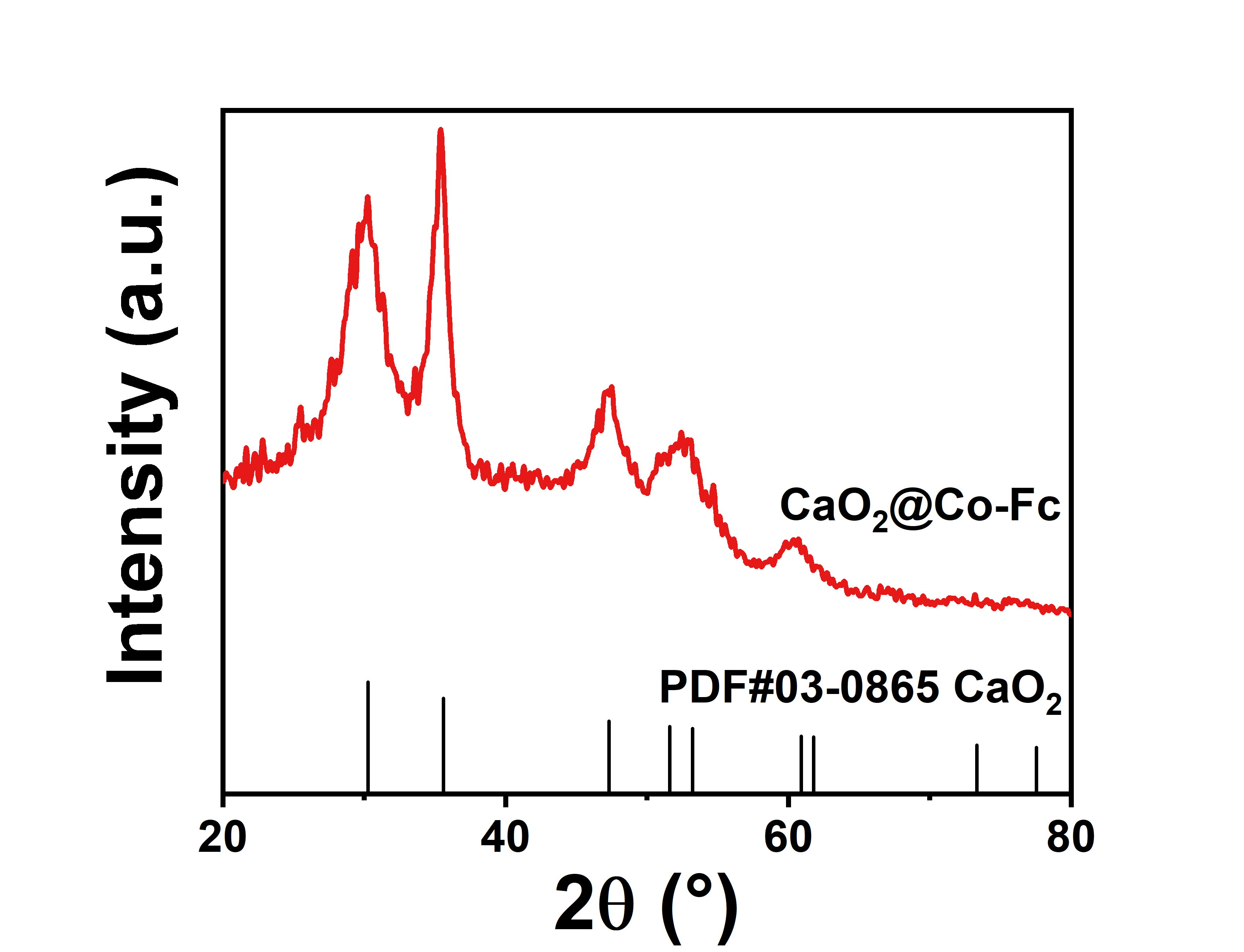


**Figure S5** XRD pattern of CaO_2_@Co-Fc.


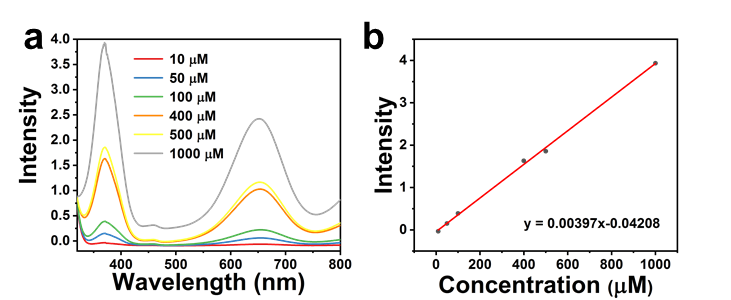


**Figure S6** Standard curves of H_2_O_2_ at the peak of 372 nm by TMB method: (a) UV-vis absorbance spectra and (b) plotting curve of TMB solution with the addition of different concentrations of H_2_O_2_.


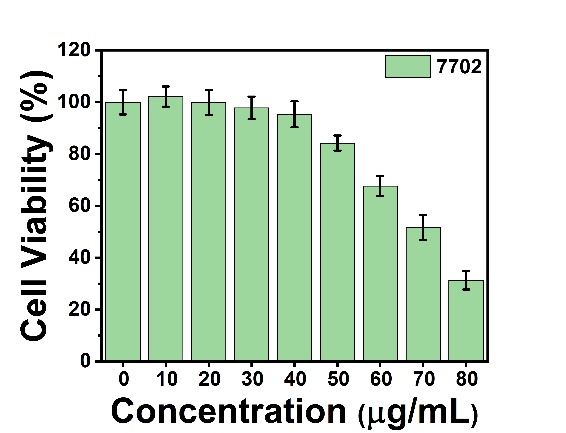


**Figure S7** The viability of 7702 cells cultured with CaO_2_ with varied concentrations.


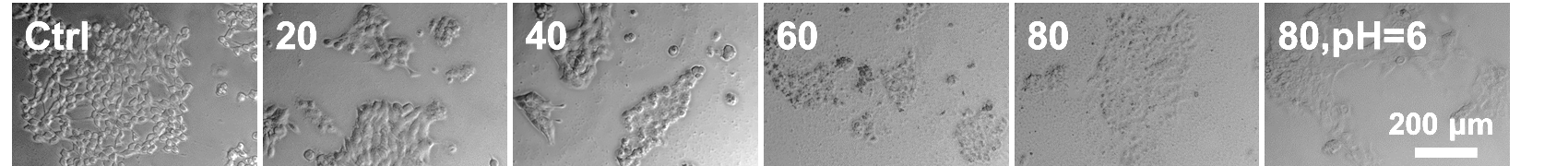


**Figure S8** Bright field images of 4T1 cells incubated with different concentrations of CaO_2_@Co-Fc after calcein-AM and PI staining for live & dead.


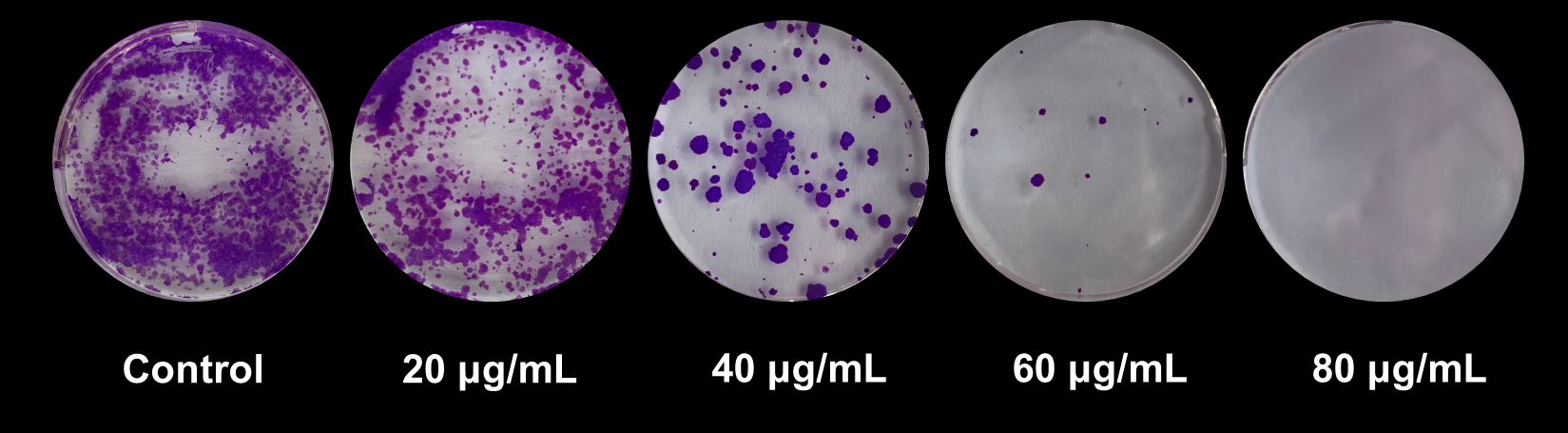


**Figure S9** Colony formation of 4T1 cells incubated with different concentrations of CaO_2_@Co-Fc.


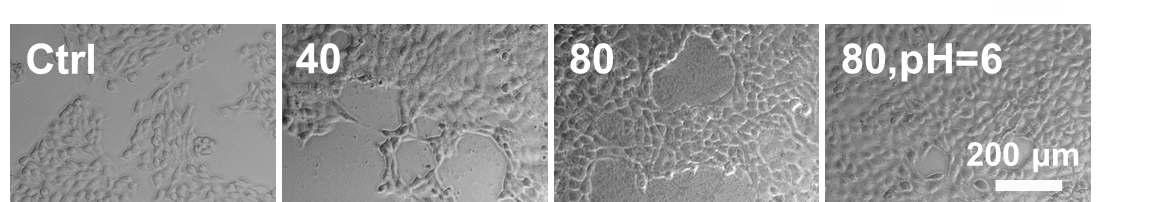


**Figure S10** Bright field images of 4T1 cells incubated with different treatments after DCFH-DA staining for intercellular ROS.


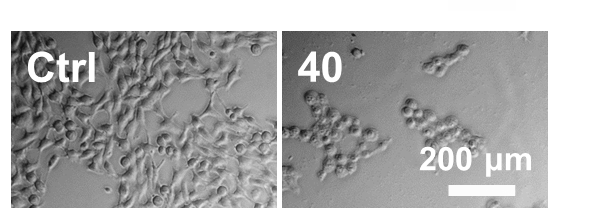


**Figure S11** Bright field images of 4T1 cells incubated with or without CaO_2_@Co-Fc after Fluo-4 AM staining for intercellular Ca^2+^ accumulation.


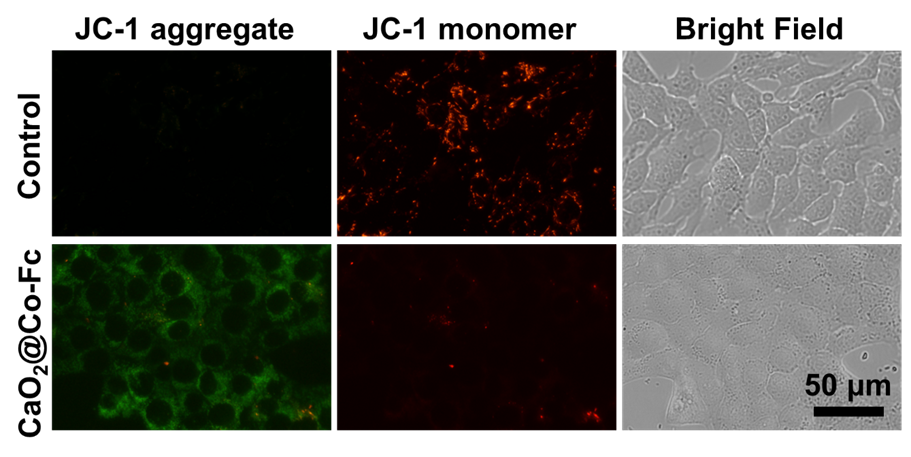


**Figure S12** Representative fluorescence images of JC-1 stained cells treated with CaO_2_@Co-Fc.


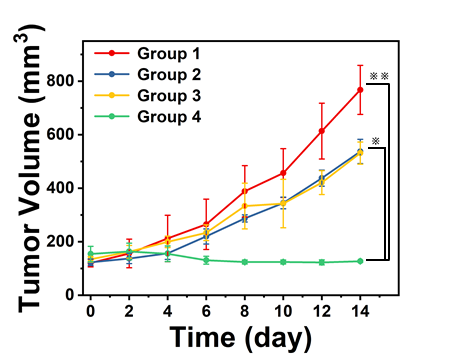


**Figure S13** Actual cancer volume variations of 4T1 tumor-bearing mice after different treatments within 14 days.
